# Exhaled Eicosanoids following Bronchial Aspirin Challenge in Asthma Patients with and without Aspirin Hypersensitivity: The Pilot Study

**DOI:** 10.1155/2012/696792

**Published:** 2012-01-12

**Authors:** L. Mastalerz, M. Sanak, J. Kumik, A. Gawlewicz-Mroczka, N. Celejewska-Wójcik, A. Ćmiel, A. Szczeklik

**Affiliations:** Department of Medicine, Medical College, Jagiellonian University, ul. Skawińska 8, 31–066 Kraków, Poland

## Abstract

*Background*. Special regulatory role of eicosanoids has been postulated in aspirin-induced asthma. *Objective*. To investigate effects of aspirin on exhaled breath condensate (EBC) levels of eicosanoids in patients with asthma. *Methods*. We determined EBC eicosanoid concentrations using gas chromatography/mass spectrometry (GC-MS) and high-performance liquid chromatography/mass spectrometry (HPLC-MS^2^) or both. Determinations were performed at baseline and following bronchial aspirin challenge, in two well-defined phenotypes of asthma: aspirin-sensitive and aspirin-tolerant patients. *Results*. Aspirin precipitated bronchial reactions in all aspirin-sensitive, but in none of aspirin-tolerant patients (ATAs). At baseline, eicosanoids profile did not differ between both asthma groups except for lipoxygenation products: 5- and 15-hydroxyeicosatetraenoic acid (5-, 15-HETE) which were higher in aspirin-induced asthma (AIA) than inaspirin-tolerant subjects. Following aspirin challenge the total levels of cysteinyl-leukotrienes (cys-LTs) remained unchanged in both groups. The dose of aspirin had an effect on magnitude of the response of the exhaled cys-LTs and prostanoids levels only in AIA subjects. *Conclusion*. The high baseline eicosanoid profiling of lipoxygenation products 5- and 15-HETE in EBC makes it possible to detect alterations in aspirin-sensitive asthma. Cysteinyl-leukotrienes, and eoxins levels in EBC after bronchial aspirin administration in stable asthma patients cannot be used as a reliable diagnostic index for aspirin hypersensitivity.

## 1. Introduction

Exhaled breath condensate (EBC) is a simple, noninvasive technique for monitoring airway inflammation. The measurement of eicosanoids in the expired breath condensate has proven to be a useful noninvasive method for the assessment and monitoring of airway inflammation in inflammatory diseases such as asthma and other pulmonary diseases [1, 2].

Eicosanoids, including prostaglandins (PGs), thromboxane A_2_ (TXA_2_), and leukotrienes (LTs), are lipid mediators involved in the pathogenesis of asthma. TXA_2_ is rapidly converted to thromboxane B_2_ (TXB_2_), a chemically stable metabolite. Thus, thromboxane synthesis in biological tissues has been monitored by measuring TXB_2_ [1, 2]. Eoxins C_4_, D_4_, and E_4_ (EXC_4_, EXD_4_, EXE_4_) are 15-lipoxygenase (15-LO) analogues of cysteinyl leukotrienes (cys-LTs). Eoxins were metabolized to eoxin E_4_ and detectable in EBC [3]. Another group of lipoxygenation products is 5-, 12- and 15-hydroxyeicosatetraenoic acid (5-HETE, 12-HETE, 15-HETE). Isoeicosanoids or isoprostanes are prostaglandin-like compounds produced by nonenzymatic lipid peroxidation of arachidonic acid. The 8-isoprostane is the best biomarker of oxidative stress and lipid peroxidation [1–3].

 A special regulatory role of eicosanoids was postulated in aspirin-induced asthma (AIA) [4–6]. AIA is characterized by nasal polyps, persistent asthma, and aspirin hypersensitivity [4]. These nasal/sinus and bronchial syndromes with aspirin hypersensitivity have been named aspirin-exacerbated respiratory disease (AERD). Indeed, in this distinct asthma phenotype [7], observations have accumulated pointing to (1) overproduction of cysteinyl-leukotrienes (cys-LTs), which are potent proinflamatory mediators and bronchoconstrictors [4, 6, 8], (2) upon ingestion of aspirin, cys-LTs are further released in rising amounts probably from eosinophils and mast cells and this is accompanied by worsening of asthmatics symptoms [9, 10], (3) depressed prostaglandin E_2_ (PGE_2_) production by peripheral blood cells [11], nasal polyps [12, 13], bronchial fibroblasts [14], diminished EP_2_ receptor on the inflammatory cells, and association with EP_2_ gene polymorphism [15, 16], (4) aspirin-precipitated asthmatic attacks are not associated with changes in the systemic prostaglandin E_2_ production, it might stem from the release of PGE_2_ from inflammatory cells during the clinical reactions to aspirin [17], (5) both prostaglandin D_2_ (PGD_2_) and its metabolite increase after aspirin-induced bronchoconstriction suggesting that this reaction is associated with mast cell activation [18–20].

Aspirin-induced changes in the levels of eicosanoids, such as prostaglandins (PGs) and cys-LTs, have been examined in various biologic samples, such as plasma [19, 20], saliva [19], induced sputum [19, 21], nasal washing fluid [22, 23], bronchoalveolar lavage [24], and urine [9, 10, 17, 25]. So far, there are few published articles on eicosanoids in EBC in aspirin-induced-asthma patients [26–28]. These studies concentrated on baseline levels of eicosanoids in EBC independent of any steroid therapy used. Interestingly, one of these studies reported prostaglandins (PGE_2_, PGF_2_ *α*, 9*α*11*β*PGF_2_) and cys-LTs levels in breath condensates in asthmatic patients after oral aspirin challenge [28]. In the present study, we focus on exhaled breath condensate concentrations of eicosanoids following bronchial aspirin challenge, local administration of aspirin in aspirin-induced asthma patients.

The aim of this study was to evaluate the changes in wide eicosanoid spectrum concentrations in EBC during asthmatic response following aspirin inhalation. We hypothesized that the profile of eicosanoids in EBC after local aspirin administrated is markedly different in aspirin-induced asthma patients as compared to asthmatics who tolerate aspirin well. These results were validated by specific analytical techniques, such as gas chromatography/mass spectrometry (GC-MS) or by high-performance liquid chromatography/mass spectrometry (HPLC-MS).

## 2. Material and Methods

### 2.1. Subjects

The study population consisted of 21 asthmatic patients sensitive to aspirin (AIA) and 23 asthmatics who tolerated aspirin well (ATA). The diagnosis of asthma was established according to GINA 2008 update. The patients' characteristics are presented in Table 1.

The diagnosis of aspirin intolerance was confirmed by oral aspirin provocation tests, performed during 36 months preceding the study. All ATA patients occasionally used aspirin without any adverse reactions. The patients had stable asthma and their baseline FEV_1_ was >70% of the predicted value on the study day. None had experienced an exacerbation or a respiratory tract infection in the 6 weeks preceding the study. Nearly 70% of ATA patients had intermittent asthma, 13% mild persistent asthma, and 17% moderate persistent. In AIA group, 67% of patients had intermittent asthma, 19% mild persistent asthma, and 14% moderate persistent asthma. According to Asthma Control Test 33% of AIA patients and 26% of ATA patients had controlled asthma, and 33% and 48% had partly controlled asthma, 33% and 26% had uncontrolled asthma, respectively. In ATA group, 2 patients were current smokers and 3 exsmokers. There were 7 exsmokers and no current smokers in AIA group. The average level of FEV_1_ and FEV_1_/FVC in AIA patients was 89.9% and 73.2%, in ATA patients was 91.5% and 78.5%, respectively.

The subjects were instructed to withhold medications that decrease bronchial responsiveness prior to aspirin challenge. Short-acting *β*_2_-agonists were not used 8 hours before the challenge. Long-acting *β*_2_-agonists and theophylline were withdrawn for 24 hours. Short-acting antihistamines and cromones were stopped 5 days before the challenge. Inhaled steroids were allowed at a dose ≤2000 *μ*g budesonide per day. None of the patients were treated with systemic corticosteroids or leukotriene modifying drugs.

Baseline and following bronchial aspirin challenge, exhaled breath condensate eicosanoids (see Table 2) levels were measured in all subjects. The patients gave informed consent and the study was approved by the University Ethics Committee.

### 2.2. Study Design

The single-blind, placebo-controlled bronchial challenge test with aspirin was carried out during one day in all study patients [29]. The test began with the inhalation of 7 breaths of placebo (saline). FEV_1_ was measured at 10 and 20 minutes after placebo inhalation. The postsaline FEV_1_ obtained at 20 minutes was used as “postsaline baseline” value.

 The consecutive doses of lysine-aspirin were inhaled every 30 minutes by increasing the concentration of lysine-aspirin and by changing the number of breaths (increasing doses of 0.18, 0.36, 0.90, 2.34, 7.20, 16.2, 39.60, 115.20 mg, at 0.5 hour intervals, up to the cumulative dose of 181.98 mg). FEV_1_ was measured at 10, 20, and 30 minutes after each dose. The challenge procedure with aspirin was interrupted, if a bronchospastic reaction occurred (FEV_1_ dropped ≥ 20%), or if the maximum cumulative dose of aspirin was reached. The cumulative dose of aspirin causing a 20% fall in FEV_1_ was calculated and recorded as PD_20_ (provocation dose of aspirin). FEV_1_ and extrabronchial symptoms were recorded at baseline, before the challenge tests, and then every 30 minutes until 6 hours after the last dose of aspirin.

In patients with positive bronchial aspirin challenge (AIA), exhaled breath condensate samples were collected for wide eicosanoid spectrum (see Table 2) estimations at baseline and at the time of appearance of the bronchial symptoms (time 0). In ATA patients, whose aspirin challenge was negative, exhaled breath condensate samples were collected at baseline and 0.5 hours after the last aspirin dose, that is, when the cumulative doses of 181.98 mg was reached (time 0).

### 2.3. Lung Function

Pulmonary function tests were performed on a flow-integrating computerized pneumotachograph (Pneumoscreen, E. Jaeger, Germany).

### 2.4. Exhaled Breath Condensate (EBC)

EBC was collected according to ATS/ERS [1] using ECO Screen instrument of Jaeger (GmbH Hoechberg, Germany). Following tidal breathing for 15–20 min, 1-2 mL of clear fluid was collected and immediately deeply frozen.

### 2.5. Biochemical Assays

Exhaled breath condensate concentration of eicosanoids was measured by gas chromatography/mass spectrometry (GC-MS) and by high-performance liquid chromatography/tandem mass spectrometry (HPLC-MS^2^) or both; see Table 2. Results of eicosanoids were recalculated as parts per million (ppm) of palmitic acid (PA) or expressed as picograms per milliliter (pg/mL). Detection limits for eicosanoids measurements were between 0.17 pg/mL for 12-HETE and 0.89 pg/mL for PGD_2_. Intraassay coefficients of variance were less than 10% and interassay coefficients of variance were less than 15%. Accuracy of measurements were better than 98.7%. Detailed analytical procedure and deuterated standards used were described elsewhere [3, 27].

### 2.6. Statistical Analysis

Summary statistics were expressed as mean (M), standard deviation (SD), median (Me), and 25% and 75% percentiles. General linear model (GLM) including repeated measures analysis of variance, which takes into account the fact that the outcome measurements are repeated over time within subject was used for multiple comparisons. Logarithmic transformation was used when needed as variance stabilizing transformation. Correlation between variables was estimated with the Spearman rank order correlations. A *P*-value ≤ 0.05 was considered statistically significant.

## 3. Results

### 3.1. Clinical Reactions

There was no statistical difference in the clinical characteristics between patients with aspirin-induced asthma (positive aspirin challenge test), and those who tolerated aspirin well (negative aspirin challenge test) except for blood eosinophil count, Table 1. None of the patients developed symptoms after administration of placebo. In aspirin-sensitive asthmatics, bronchial reactions developed after 0.18 mg in 1 subject, after 0.36 mg in 2 subjects, after 0.9 mg in 3 subjects, following 2.34 mg in 2, after 7.2 mg in 5 subjects, after 16.2 mg in 4 subjects, following 39.6 mg in 2 subjects, and after 115.2 mg in 2 subjects. The mean cumulative dose of aspirin was 32.108 mg. All the symptoms were relieved by short-acting *β*_2_-agonists. None of ATA patients developed any clinical symptoms following aspirin challenges. 

#### 3.1.1. Cyclooxygenase Products

**Exhaled Breath Condensate Concentration of Prostanoids**

At baseline (Table 2), exhaled breath condensate levels of PGD_2_, such as its metabolite 9*α*11*β*PGF_2_, and PGF_2_ *α*, 6-keto-PGF_1_ *α* and nonenzymatic isomer (8-iso-PGF_2_), did not differ significantly between the study groups. Statistically, results did not differ dependently on the methods used (HPLC/MS/MS or GC/MS) for marking eicosanoids and results shown (ppm of PA or pg/mL).

Following aspirin administration, no significant differences in EBC levels of PGD_2_ measured by HPLC/MS/MS (results were recalculated as ppm of PA) and 9*α*11*β*PGF_2_ measured by GC/MS (results were expressed as ppm of PA) were found in AIA (*P* = 0.13 and *P* = 0.82, resp.) and in ATA subjects (*P* = 0.69 and *P* = 0.43, resp.). The same marked result (no differences) was observed when PGD_2_ was measured by GC-MS.

In cases when 11-dehydro TXB_2_ resulted as ppm of PA, at baseline (Table 2) and following aspirin challenge (ANOVA, *P* = 0.66) exhaled breath condensate level of 11-dehydro TXB_2_ did not differ significantly between the study groups.

Negative correlation was founded between provocation doses of aspirin and exhaled PGD_2_ and its 9*α*11*β*PGF_2_, PGF_2_ *α*, 6-keto-PGF_1_ *α*, and 11-dehydro TXB_2_ levels only in AIA patients.

#### 3.1.2. Lipoxygenation Products

**Exhaled Breath Condensate Concentration of Cysteinyl-Leukotriene (Cys-LTs)**

At baseline (Table 2), exhaled breath condensate levels of leukotrienes C_4_, D_4_, and E_4_ did not differ significantly between the AIA and ATA groups (*P* = 0.43, *P* = 0.22, *P* = 0.79, resp.).

In both study groups, following aspirin challenge, EBC level of LTC_4_ decreased significantly (ANOVA, *P* = 0.003). No significant differences in EBC levels of LTD_4_ was found in AIA and in ATA group patients (ANOVA, *P* = 0.67), where the level remained unchanged and at a constant. Exhaled LTE_4_ after aspirin challenge increased significantly in both study groups (ANOVA, *P* = 0.03). However, total level of cys-LTs (the sum of LTC4, LTD4, LTE4) showed no changes in either group studied. Statistically the results of cys-LTs in EBC were identical independent of units (measurement) used (ppm of PA or pg/mL).

The dose of inhaled steroid used by study patients and FEV_1_ values had no effect on magnitude of the response of the cys-LTs and its duration.

Negative correlation was founded between provocation doses of aspirin and exhaled LTC_4_ (*r* = −0.47, *P* = 0.04), LTD_4_ (*r* = −0.46, *P* = 0.04) and LTE_4_ (*r* = −0.43, *P* = 0.05) levels only in AIA patients.

**Exhaled Breath Condensate Concentration of Leukotriene B_4_**

At baseline (Table 2), exhaled breath condensate level of leukotriene B_4_ did not differ significantly between the AIA and ATA groups (*P* = 0.36).

Following aspirin administration, EBC levels of LTB_4_ decreased significantly only in aspirin-sensitive patients (ANOVA, *P* = 0.03). No differences were observed when LTB_4_ was expressed as pg/mL.

**Exhaled Breath Condensate Concentration of HETE**

**Figure 1:**
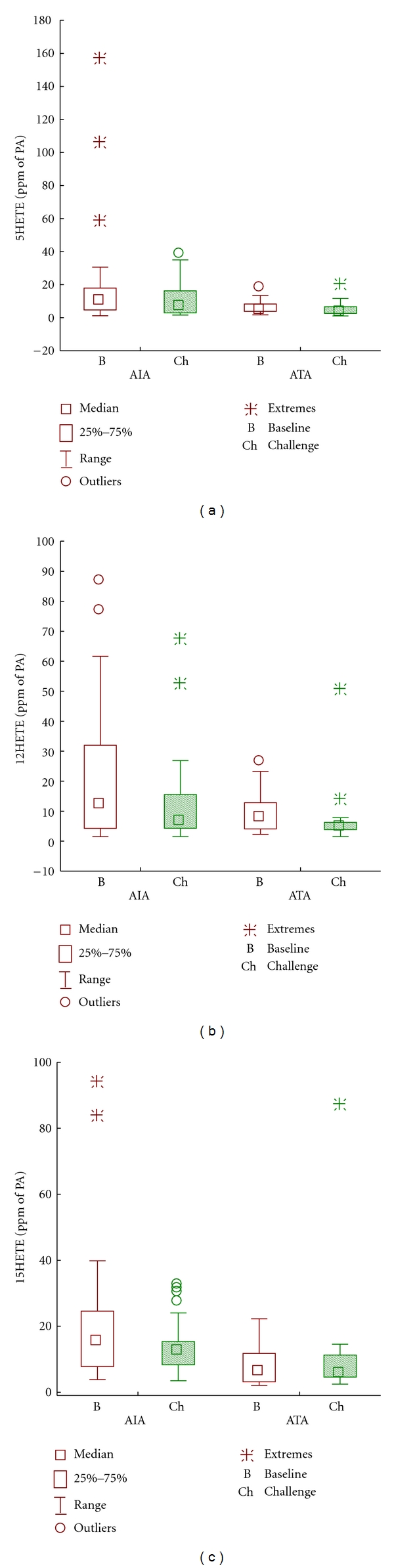
Eicosanoids' levels in exhaled breath condensate before and after bronchial aspirin challenge. (a) 5-HETE, (b) 15-HETE, and (c) 12-HETA. AIA: aspirin-induced asthma. ATA: aspirin-tolerant asthma. B: baseline. Ch: challenge.

At baseline (Table 2), exhaled breath condensate levels of 5-HETE and 15-HETE were significantly higher in AIA as compared to ATA groups (*P* = 0.03, *P* = 0.001, resp.).  Figure 1. Following bronchial aspirin administration, EBC levels of 5- and 15-HETE (ANOVA, *P* = 0.23) remained unchanged in AIA (*P* = 0.37 and *P* = 0.23, resp.) and ATA patients.

At baseline (Table 2), exhaled breath condensate levels of 12-HETE did not differ significantly between the AIA and ATA groups (*P* = 0.13). Following aspirin administration, EBC levels of 12-HETE decreased significantly only in ATA group (*P* = 0.03, due to outliers Wilcoxon Matched pairs test was used).

The dose of inhaled steroid used by patients and FEV_1_ values had no effect on magnitude of the response of the 5- and 15-HETE. At baseline, negative correlation was found between the doses of steroids and EBC levels of 12-HETE only in aspirin-sensitive subjects (*r* = −0.45, *P* = 0.04).

The dose of aspirin, had no effect on the magnitude of response of 5- and 15-HETE.

**Exhaled Breath Condensate Concentration of Eoxins**

At baseline and following aspirin challenge, exhaled breath condensate levels of eoxins C_4_, D_4_, and E_4_ did not differ significantly between the AIA and ATA groups (see Table 2).

At baseline and following aspirin challenge no correlation was found between provocation doses of aspirin, inhaled steroid therapy and FEV_1_ values, and eoxins EBC levels in aspirin-sensitive and aspirin-intolerant patients.

## 4. Discussion

In this study, we used a validated analytic platform [27, 30, 31] to analyze eicosanoids in EBC of asthmatic patients. A highly sensitive method of gas chromatography/mass spectrometry or high-performance liquid chromatography/mass spectrometry or both were used to measure spectrum of eicosanoids-nonvolatile compounds present in EBC [3, 27, 31]. Novel concepts for the standardization of EBC material measurements have been introduced to obtain characteristics of eicosanoid patterns produced by asthmatic lungs [27]. The assessment of palmitic acid content in EBC among many other methods [32] seems to be a convenient solution for compensating the “dilution factor” [3]. It has recently been demonstrated that dilution of nonvolatile compounds in water differs between the subjects by more than 1 order of magnitude and depends on ventilation mechanics [33, 34]. For that reason, in this paper, data are recalculated as parts per million of palmitic acid. For better understanding, EBC eicosanoid levels are given in both applicable units: ppm of PA (Table 2) and in pg/mL (Table 3) independently of performed assay.

The aim of this study was to compare a wide profile of eicosanoids released to the epithelial surface of the asthmatic lung in subjects with and without aspirin hypersensitivity at baseline and following bronchial aspirin challenge, most of them on chronic inhaled steroid therapy. We demonstrated for the first time exhaled eicosanoids following local administration of aspirin in aspirin-induced asthma patients.

Comparing subjects with AIA and ATA no significant differences were observed in EBC levels of cyclooxygenase pathway prostanoid products (PGD_2_ and its metabolite 9*α*11*β*PGF_2_, PGF2*α*, 6-keto-PGF1*α*, and 11-dehydro TXB_2_) and nonenzymatic isomer (8-iso-PGF_2_) in neither baseline nor after aspirin inhalation. Opposing data has been earlier reported as lower [28] and other times higher [27] baseline PGD_2_ metabolite, namely, 9*α*,11*β*PGF_2_, levels in EBC in AIA patients. Differences observed in this previous study between both asthma phenotypes [27] can possibly be explained by lower FEV_1_ values and more severe of disease in patients with aspirin hypersensitivity compared to subjects who tolerated aspirin well. Moreover, a significant predictor of decreased FEV_1_ was increased 9*α*,11*β*-PGF_2_ only in AIA which did not correlate in ATA subjects [27]. In our study, all patients presented comparable FEV_1_ values, and most likely, similar bronchoconstricting eicosanoid levels such as PGD_2_ and its metabolite. Mast cells are probably the main source of PGD_2_ overproduction. Higher global production of PGD_2_ metabolite 9*α*,11*β*PGF_2_ was also present in the blood and urine at baseline in AIA subjects [18, 20]. Concentration of 9*α*,11*β*-PGF_2_ was not changed by the oral-systemic [28] and how indicated our data bronchial-local administration of aspirin.

Our results revealed a significant upregulation of some arachidonate lipoxygenation products in asthmatic subjects with aspirin hypersensitivity, as manifested by high baseline levels of 5-, 15-HETE in EBC. This data is consistent with the latest observations [27]. These findings are related to an overexpression of lipoxygenases enzymes, particularly 5- and 15-LO in the asthmatic lung with aspirin hypersensitivity. These enzymes are expressed in eosinophils, activated macrophages, and also in lymphocytes and mast cells. Kowalski and colleagues have demonstrated that aspirin triggers specific generation of 15-HETE from nasal polyp epithelial cells [12] and peripheral blood leukocytes [35, 36] from aspirin-sensitive but not aspirin-tolerant subjects with asthma/rhinosinusitis. Also, they have demonstrated that two alternatively spliced variants of COX-1 mRNA present in human leucocytes may be differently expressed in patients with asthma. The relative expression of those variants has been correlated to aspirin-triggered 15-HETE generation suggesting association of this phenomenon with the pathogenesis of aspirin-induced asthma [37].

No elevation of baseline 12-HETE in EBC comparing AIA and ATA subjects was observed. Whereas, after bronchial aspirin administration, there was a statistically significant decrease in 12-HETE concentration noted only in ATA subjects. On that basis, we assume blood platelets, the main source of 12-LO, may possibly play some role in pathogenesis of aspirin hypersensitivity. However, 12-LO was originally cloned from respiratory epithelia, where 15-LO activity was also found [38].

Our study did not demonstrated baseline local overproduction of cys-LTs in the airways in AIA and is consistent with an earlier study [28]. Baseline EBC levels of LTC_4_, LTD_4_, and LTE_4_ did not differ between aspirin tolerant and hypersensitive subjects. Contrary to this finding in subjects with AIA, a higher baseline level of cys-LTs in EBC was also reported [26, 27]. This might have been a result of low FEV_1_ values and minor control of asthma (severity index) [27] or steroid-naive [26] in aspirin hypersensitivities compared to aspirin tolerant subjects. The levels of exhaled cys-LTs were lower in those AIA subjects who received inhaled steroid therapy [26]. Following bronchial aspirin challenge levels of particular cys-LTs showed some variations but the total concentration of cys-LTs remained unchanged in both study groups. Up till now, cys-LTs level in EBC has not been measured after local (inhaled) aspirin administration. However, it has been shown [28] that cys-LTs levels in EBC after oral (systemic administration) aspirin challenge increased significantly in subjects with AIA. Varying results of different studies implicate that levels of cys-LTs in EBC cannot be a convenient indicator of asthma phenotype as their level after challenge test possibly depends on manner of aspirin administration and probably the doses of steroids inhaled. Results can be surprising because, a key enzyme—LTC4 synthase, is overexpressed in bronchial mucosa of patient with AIA [39]. Furthermore, circulating steroid blood eosinophils—main source of LTE_4_—carry more mRNA transcripts for this enzyme [40].

As was reported in childhood asthma [41] and adult asthmatics [27], 15-LO analogues of cys-LTs, eoxins C_4_, D_4_, and E_4_, showed no increase at baseline in asthmatic subjects independent of aspirin hypersensitivity. Their concentrations were not changed by the bronchial aspirin challenge. Role of those eicosanoids has been recently investigated in aspirin hypersensitive patients.

The high-sensitivity eicosanoid profiling of lipoxygenation products (5HETE, 15HETE) in EBC makes it possible to detect alterations in asthma, especially in its distinct phenotype characterized by hypersensitivity to aspirin and other nonsteroidal anti-inflammatory drugs. Cysteinyl-leukotriene levels in EBC after aspirin challenge in stable asthma patients, not steroid naive, most probably cannot be used as a reliable and sensitive index for aspirin hypersensitivity. In stable AIA patients on chronic inhaled steroid therapy of global (urinary) rather than in local (breath condensate) production of postchallenge cys-LTs is of greater and more sensitive value for aspirin hypersensitivity. We believe that quantitate cell analysis and measurements of released eicosanoides in induced-sputum will be more applicable for that purpose.

## Figures and Tables

**Table 1:** Clinical characteristics of the patients.

	AIA (*n* = 21)	ATA (*n* = 23)	*P*
Age (y)	44.9 ± 13.96 39 (34 ÷ 58)	39.8 ± 8.71 39 (32 ÷ 45)	N.S. (0.33)
Female/Male	10/11	15/8	N.S. (0.36)
Duration of asthma (y)	12.36 ± 8.07 13 (6÷18)	10.26 ± 11.33 7 (1 ÷ 17)	N.S. (0.19)
Inhaled steroids yes/no	18/3	22/1	N.S. (0.34)
Inhaled steroids (*μ*g/d) flutikason	616.57 ± 425.83 500 (500 ÷ 1000)	726.09 ± 667.06 500 (250 ÷ 1000)	N.S. (0.93)
FEV_1_ baseline (% predicted) placebo/aspirin day	89.87 ± 10.83 90.01 (83.45 ÷ 95.29)	91.45 ± 11.47 91.3 (84.81 ÷ 102.1)	N.S. (0.57)
Total IgE (IU/mL)	115.06 ± 111.41 76.3 (42.8 ÷ 158)	162.86 ± 177.82 66.8 (24.1 ÷ 179)	N.S. (0.78)
Skin prick test (*n*) positive/negative	10/9	15/8	N.S. (0.53)
Blood eosinophil count	472.67 ± 295.39* 424 (324 ÷ 513)	248.22 ± 173.32* 232 (119 ÷ 309)	0.003

Values are expressed as mean ± SD, and median (25% and 75% percentiles).

AIA: aspirin-induced asthma.

ATA: aspirin-tolerant asthma.

**Table 2:** Eicosanoids values at baseline and following aspirin challenge in AIA and ATA patients. Results of eicosanoids were recalculated as parts per million (ppm) of palmitic acid (PA).

	AIA (*n* = 21)		ATA (*n* = 23)		*P* (ANOVA)
	Baseline	Challenge	Baseline	Challenge	After the challenge
PGD_2_	5.22 ± 5.93	4.85 ± 3.92	4.71 ± 3.38	4.36 ± 2.83	0.54
(parts/million of PA)	3.18	3.69	4.27	3.85	
GC/MS	(1.35 ÷ 5.85)	(1.56 ÷ 6.99)	(2.02 ÷ 6.37)	(1.78 ÷ 6.31)	

PGD_2_	4.65 ± 3.14	3.76 ± 2.91	3.85 ± 2.06	3.72 ± 1.58	Unable
(parts/million of PA)	3.56	3.00	2.99	3.33	
HPLC/MS/MS	(1.93 ÷ 7.02)	(1.81 ÷ 5.66)	(2.11 ÷ 5.38)	(2.47 ÷ 4.83)	

9*α*11*β*PGF_2_	2.20 ± 2.01	2.21 ± 2.02	0.73 ± 0.39	0.79 ± 0.44	Unable
(parts/million of PA)	1.24	1.30	0.61	0.75	
GC/MS	(0.27 ÷ 4.34)	(0.28 ÷ 3.90)	(0.45 ÷ 1.05)	(0.45 ÷ 1.02)	

PGF2*α*	1.67 ± 1.59	1.41 ± 1.38	1.14 ± 1.26	0.99 ± 1.18	0.82
(parts/million of PA)	0.97	0.86	0.64	0.49	
GC/MS	(0.26 ÷ 2.71)	(0.42 ÷ 2.16)	(0.43 ÷ 1.16)	(0.38 ÷ 1.38)	

6-keto-PGF1*α*	30.23 ± 18.50	29.27 ± 16.26	27.98 ± 24.71	29.64 ± 27.20	0.74
(parts/million of PA)	32.15	32.96	16.80	16.12	
GC/MS	(11.76 ÷ 45.67)	(17.09 ÷ 37.44)	(12.20 ÷ 39.60)	(13.82 ÷ 41.81)	

6-keto-PGF1*α*	30.51 ± 19.45	28.92 ± 16.65	26.79 ± 23.71	28.77 ± 25.48	0.11
(parts/million of PA)	32.08	32.51	15.60	18.15	
HPLC/MS/MS	(11.13 ÷ 44.05)	(15.78 ÷ 38.12)	(11.91 ÷ 35.68)	(13.20 ÷ 28.65)	

11-dehydro TXB2	19.34 ± 10.77	20.16 ± 11.23	16.43 ± 8.14	17.72 ± 9.79	0.82
(parts/million of PA)	21.67	21.40	15.10	15.55	
GC/MS	(12.18 ÷ 28.14)	(12.25 ÷ 26.23)	(9.77 ÷ 21.96)	(11.74 ÷ 17.17)	

11-dehydro TXB2	18.86 ± 10.82	20.50 ± 11.95	16.15 ± 8.20	16.87 ± 8.81	0.66
(parts/million of PA)	19.69	21.48	14.49	14.65	
HPLC/MS/MS	(9.18 ÷ 27.50)	(11.26 ÷ 25.83)	(9.37 ÷ 22.04)	(10.97 ÷ 19.78)	

LTC_4_	14.51 ± 15.87	15.57 ± 36.94*	9.76 ± 14.06	4.16 ± 3.08*	0.003*
(parts/million of PA)	9.22	6.61	5.40	3.24	
HPLC/MS/MS	(2.18 ÷ 22.31)	(2.08 ÷ 11.50)	(2.79 ÷ 10.84)	(1.65 ÷ 5.84)	

LTD_4_	3.11 ± 2.80	2.68 ± 2.21	3.31 ± 2.59	2.57 ± 1.55	0.67
(parts/million of PA)	2.18	2.25	2.68	2.16	
HPLC/MS/MS	(0.83 ÷ 4.41)	(1.22 ÷ 3.53)	(1.60 ÷ 4.41)	(1.42 ÷ 3.58)	

LTE_4_	6.14 ± 3.79	11.96 ± 19.51*	5.45 ± 2.83	6.47 ± 2.57*	0.03*
(parts/million of PA)	5.19	6.38	4.88	6.54	
HPLC/MS/MS	(3.23 ÷ 8.50)	(4.95 ÷ 14.57)	(3.10 ÷ 7.76)	(4.42 ÷ 7.59)	

Total cysLTs	23.40 ± 19.22	30.54 ± 56.90	18.51 ± 15.42	13.20 ± 5.41	0.33
(parts/million of PA)	19.74	15.70	14.14	12.94	
HPLC/MS/MS	(6.67 ÷ 31.22)	(6.68 ÷ 29.20)	(10.95 ÷ 21.25)	(8.17 ÷ 18.75)	

LTB_4_	154.84 ± 187.68	73.88 ± 77.38*	101.37 ± 163.65	70.69 ± 73.37	0.03*
(parts/million of PA)	70.28	50.49	49.72	32.29	
HPLC/MS/MS	(24.57 ÷ 168.26)	(28.71 ÷ 86.75)	(19.05 ÷ 126.82)	(19.01 ÷ 91.91)	

5 HETE	23.95 ± 39.08*	11.21 ± 11.26	6.08 ± 3.83*	5.41 ± 4.41	Unable
(parts/million of PA)	9.98	6.78	4.53	4.22	
HPLC/MS/MS	(4.71 ÷ 17.95)	(2.92 ÷ 16.24)	(3.82 ÷ 8.32)	(2.63 ÷ 6.68)	

12 HETE	23.58 ± 25.35	14.58 ± 17.15	9.22 ± 6.48	7.17 ± 9.85*	Unable
(parts/million of PA)	12.49	6.62	7.93	5.23	
HPLC/MS/MS	(4.27 ÷ 32.00)	(4.31 ÷ 15.53)	(4.11 ÷ 12.84)	(3.87 ÷ 6.26)	

15 HETE	22.65 ± 24.08*	14.84 ± 9.22	7.73 ± 5.52*	10.47 ± 17.07	0.23
(parts/million of PA)	15.48	12.80	6.72	6.13	
HPLC/MS/MS	(7.80 ÷ 24.57)	(8.35 ÷ 15.39)	(3.17 ÷ 11.75)	(4.62 ÷ 11.25)	

EXC_4_	2.72 ± 2.45	2.99 ± 3.08	2.00 ± 1.67	1.99 ± 1.69	0.79
(parts/million of PA)	2.09	2.59	1.41	1.61	
HPLC/MS/MS	(1.17 ÷ 3.42)	(0.65 ÷ 4.32)	(1.09 ÷ 2.38)	(0.68 ÷ 2.38)	

EXD_4_	2.98 ± 2.21	2.77 ± 2.52	3.03 ± 2.50	2.87 ± 2.73	0.69
(parts/million of PA)	2.12	1.97	2.57	2.22	
HPLC/MS/MS	(0.82 ÷ 4.57)	(1.12 ÷ 4.13)	(0.90 ÷ 4.09)	(0.97 ÷ 4.46)	

EXE_4_	7.86 ± 5.11	8.48 ± 16.15	8.09 ± 9.65	5.65 ± 5.28	0.95
(parts/million of PA)	7.06	3.82	3.79	3.65	
HPLC/MS/MS	(4.00 ÷ 11.43)	(2.89 ÷ 7.03)	(2.67 ÷ 12.23)	(2.33 ÷ 8.97)	

8-iso-PGF2*α*	0.73 ± 0.40	0.79 ± 0.38	0.72 ± 0.26	0.82 ± 0.32	0.09
(parts/million of PA)	0.68	0.76	0.67	0.73	
GC/MS	(0.40 ÷ 1.04)	(0.54 ÷ 1.11)	(0.58 ÷ 0.83)	(0.55 ÷ 1.08)	

Median (25% and 75% percentiles).

AIA: aspirin-induced asthma. ATA: aspirin-tolerant asthma. PA: palmitic acid.

*P-values < 0.05; AIA versus ATA at baseline or after the challenge.

*P*-values:

* AIA versus ATA at baseline.

*baseline versus challenge in AIA.

*baseline versus challenge in ATA.

**Table 3:** Eicosanoids values at baseline and following aspirin challenge in AIA and ATA patients. Results of eicosanoids were recalculated as picograms per milliliter (pg/mL).

	AIA (*n* = 21)		ATA (*n* = 23)		*P* (ANOVA)
	Baseline	After the challenge	Baseline	After the challenge	After the challenge
PGD_2_	1.49 ± 1.17	1.54 ± 1.02	2.35 ± 2.06	1.80 ± 1.22	0.35
(pg/mL)	1.01	1.21	1.64	1.53	
GC/MS	(0.82 ÷ 1,68)	(0.79 ÷ 2.11)	(1.09 ÷ 2.70)	(0.85 ÷ 3.20)	

PGD_2_	1.53 ± 0.81	1.14 ± 0.58	1.76 ± 1.14	1.63 ± 0.93	0.05
(pg/mL)	1.39	1.09	1.48	1.45	
HPLC	(0.93 ÷ 2.02)	(0.67 ÷ 1.36)	(0.99 ÷ 2.19)	(0.80 ÷ 2.07)	

9*α*11*β*PGF_2_	0.48 ± 0.33	0.50 ± 0.34	0.32 ± 0.18	0.31 ± 0.17	0.59
(pg/mL)	0.36	0.34	0.29	0.28	
GC/MS	(0.16 ÷ 0.69)	(0.25 ÷ 0.75)	(0.20 ÷ 0.35)	(0.21 ÷ 0.38)	

PGF2*α*	0.42 ± 0.34	0.40 ± 0.35	0.43 ± 0.33	0.39 ± 0.45	0.82
(pg/mL)	0.28	0.27	0.30	0.23	
GC/MS	(0.19 ÷ 0.56)	(0.15 ÷ 0.59)	(0.23 ÷ 0.44)	(0.16 ÷ 0.35)	

6-keto-PGF1*α*	8.94 ± 4.30	8.70 ± 3.72	9.95 ± 4.54	9.55 ± 4.19	0.53
(pg/mL)	7.17	7.22	7.48	7.46	
GC/MS	(6.75 ÷ 7.79)	(7.02 ÷ 7.62)	(7.24 ÷ 15.02)	(7.24 ÷ 11.49)	

6-keto-PGF1*α*	8.99 ± 4.41	8.59 ± 3.93	9.69 ± 4.37	9.46 ± 3.95	0.60
(pg/mL)	7.26	6.96	7.40	7.65	
HPLC/MS/MS	(6.32 ÷ 8.41)	(6.59 ÷ 8.54)	(7.08 ÷ 13.54)	(7.13 ÷ 10.27)	

11-dehydro TXB2	5.74 ± 0.87*	5.94 ± 0.89	6.52 ± 0.92*	6.39 ± 0.94	0.18
(pg/mL)	5.74	6.17	6.81	6.62	
GC/MS	(5.14 ÷ 6.34)	(5.16 ÷ 6.49)	(6.35 ÷ 7.00)	(5.70 ÷ 7.16)	

11-dehydro TXB2	5.51 ± 0.90*	5.96 ± 1.11	6.40 ± 1.17*	6.18 ± 1.01	0.03*
(pg/mL)	5.49	6.03	6.63	6.18	
HPLC/MS/MS	(4.77 ÷ 6.16)	(5.24 ÷ 6.55)	(6.01 ÷ 7.03)	(5.55 ÷ 7.09)	

LTC_4_	4.17 ± 4.56	3.69 ± 7.01	4.35 ± 4.57	1.87 ± 1.73*	0.01*
(pg/mL)	2.30	2.02	2.46	1.06	
HPLC/MS/MS	(1.23 ÷ 6.42)	(1.13 ÷ 3.36)	(1.05 ÷ 6.18)	(0.59 ÷ 2.74)	

LTD_4_	0.88 ± 0.60	0.85 ± 0.59	1.58 ± 1.28	1.18 ± 0.88	0.16
(pg/mL)	0.69	0.65	1.34	0.95	
HPLC/MS/MS	(0.43 ÷ 1.19)	(0.50 ÷ 1.03)	(0.55 ÷ 2.04)	(0.41 ÷ 1.77)	

LTE_4_	2.03 ± 0.92	3.20 ± 3.63*	2.46 ± 1.43	2.78 ± 1.44*	0.04*
(pg/mL)	1.83	2.33	1.93	2.45	
HPLC/MS/MS	(1.28 ÷ 2.71)	(1.57 ÷ 3.44)	(1.30 ÷ 3.45)	(1.54 ÷ 3.69)	

Total cysLTs	7.30 ± 4.98	8.02 ± 10.75	8.39 ± 5.62	5.83 ± 3.40	0.24
(pg/mL)	5.97	6.01	7.24	4.90	
HPLC/MS/MS	(4.14 ÷ 9.66)	(3.91 ÷ 6.75)	(3.06 ÷ 12.75)	(2.87 ÷ 8.87)	

LTB_4_	69.09 ± 102.42	26.15 ± 25.60*	54.23 ± 99.10	34.81 ± 37.83	0.02*
(pg/mL)	29.66	16.99	27.73	15.35	
HPLC/MS/MS	(10.26 ÷ 50.00)	(10.41 ÷ 30.38)	(6.40 ÷ 57.18)	(6.37 ÷ 53.27)	

5 HETE	7.15 ± 9.80	3.60 ± 2.69	2.75 ± 1.94	2.61 ± 2.99	0.79
(pg/mL)	2.94	3.41	2.29	1.67	
HPLC/MS/MS	(1.96 ÷ 7.41)	(1.67 ÷ 5.29)	(1.43 ÷ 3.12)	(0.78 ÷ 3.00)	

12 HETE	6.64 ± 6.16	5.85 ± 10.45	4.03 ± 2.68	3.45 ± 6.75*	0.02*
(pg/mL)	4.05	2.82	3.10	1.93	
HPLC/MS/MS	(2.91 ÷ 7.76)	(1.52 ÷ 4.22)	(1.53 ÷ 5.84)	(1.25 ÷ 2.85)	

15 HETE	6.99 ± 4.87*	5.81 ± 5.14	3.48 ± 2.78*	5.29 ± 11.76	0.30
(pg/mL)	5.96	4.77	2.95	2.39	
HPLC/MS/MS	(4.04 ÷ 8.27)	(2.53 ÷ 6.52)	(1.75 ÷ 3.98)	(1.92 ÷ 2.97)	

EXC_4_	1.05 ± 0.82	0.92 ± 0.89	0.89 ± 0.57	0.83 ± 0.73	0.74
(pg/mL)	0.79	0.59	0.80	0.61	
HPLC/MS/MS	(0.45 ÷ 1.61)	(1.18 ÷ 0.58)	(0.36 ÷ 1.40)	(0.25 ÷ 1.07)	

EXD_4_	1.71 ± 2.29	1.78 ± 2.82	1.70 ± 1.76	1.29 ± 1.54	0.909
(pg/mL)	0.73	0.62	1.06	0.74	
HPLC/MS/MS	(0.30 ÷ 1.51)	(0.24 ÷ 1.93)	(0.27 ÷ 2.31)	(0.39 ÷ 1.96)	

EXE_4_	5.47 ± 8.61	3.68 ± 6.01	4.01 ± 4.97	2.58 ± 2.63	0.07
(pg/mL)	2.15	1.05	1.59	1.35	
HPLC/MS/MS	(0.97 ÷ 4.60)	(0.71 ÷ 2.66)	(0.89 ÷ 5.66)	(0.72 ÷ 3.89)	

8-iso-PGF2*α*	0.25 ± 0.12*	0.28 ± 0.21	0.33 ± 0.15*	0.32 ± 0.10	0.92
(pg/mL)	0.20	0.21	0.35	0.34	
GC/MS	(0.17 ÷ 0.28)	(0.19 ÷ 0.26)	(0.20 ÷ 0.42)	(0.25 ÷ 0.39)	

Median (25% and 75% percentiles).

AIA: aspirin-induced asthma. ATA: aspirin-tolerant asthma.

*P-values < 0.05; AIA versus ATA at baseline or after the challenge.

*P*-values:

* AIA versus ATA at baseline.

*baseline versus challenge in AIA.

*baseline versus challenge in ATA.

## List of Abbreviations

AIA: Aspirin-intolerant asthma
ATA: Aspirin-tolerant asthma
EBC: Exhaled breath condensate
GC-MS: Gas chromatography-mass spectrometry
HPLC-MS/MS: High-performance liquid chromatography-tandem mass spectrometry
COX: Cyclooxygenase
PG: Prostaglandin
9*α*,11*β*-PGF_2_: PGD_2_ semistable metabolite
6-keto-PGF_1_*α*: Prostacyclin metabolite
11-dehydro-TXB2: Thromboxane A2 metabolite
EP2: Prostaglandin receptor 2
LO: Lipoxygenase
HETE: Hydroxyeicosatetraenoic acid
LT: Leukotriene
Cys-LTs: Cysteinyl leukotrienes
8-iso-PGF_2_*α*: Isoprostane F_2_ *α*
NSAIDs: Nonsteroidal anti-inflammatory drugs.
